# Investigating the Role of Sodium Butyrate in Mouse Models of IBD: A Multimodal Structural and Functional MRI Study

**DOI:** 10.1155/np/9003986

**Published:** 2026-09-09

**Authors:** Ran Fan, Siqi Li, Cuiping Bao, Weiwei Cui, Li Yu, Hong Yu, Xuehuan Liu, Jun Liu

**Affiliations:** ^1^ Department of Radiology, Tianjin Union Medical Center, The First Affiliated Hospital of Nankai University, Tianjin 300121, China, tijmu.edu.cn; ^2^ School of Medicine, Nankai University, Tianjin 300071, China, nankai.edu.cn; ^3^ Tianjin University of Traditional Chinese Medicine, Tianjin 301617, China, tjutcm.edu.cn; ^4^ Tianjin Fourth Central Hospital, The Affiliated Hospital of Tianjin Medical University, Tianjin 300140, China; ^5^ Department of Rehabilitation Medicine, Binzhou Medical University Hospital, Binzhou 256600, Shandong Province, China, bzmc.edu.cn; ^6^ Tianjin Fourth Central Hospital, Tianjin University, Tianjin 300140, China, tju.edu.cn

**Keywords:** brain, butyrate, gut–brain axis, inflammatory bowel disease, magnetic resonance imaging

## Abstract

Patients with inflammatory bowel disease (IBD) experience abnormalities in the central nervous system via the gut–brain axis (GBA), leading to changes in brain structure and function. Butyrate has been shown to influence the central nervous system via the GBA, with potential benefits for neurological issues in IBD. However, directly assessing butyrate’s impact on the central nervous system in IBD patients is difficult due to confounding factors in human studies. This study employed a mouse model to investigate the effects of sodium butyrate (NaB) on brain structure and function in IBD through multimodal MRI. Fifteen mice were equally divided into three groups: a healthy control group, an IBD group and an IBD + NaB group (NaB group). All three groups underwent scanning with a 9.4T magnetic resonance imaging scanner. The scanning sequences included diffusion tensor imaging (DTI), three‐dimensional T2‐weighted imaging and functional MRI (fMRI), respectively, to assess alterations in white matter integrity, grey matter nucleus volume and neural activity among the different groups. Compared with the IBD group, the NaB group exhibited reduced inflammation. In both the IBD and NaB groups, the mean diffusivity (MD) values of white matter fibre tracts in the left inferior cerebellar peduncle (restiform body) region were lower than those in the HC group. Differences in nucleus volume were observed among the groups in two regions: an undefined region and the left caudoputamen. Differences in neural activity were observed among the groups in the right pontine reticular nucleus, lateral posterior nucleus of the thalamus, left simple lobule and right midbrain reticular nucleus. Furthermore, alterations in neural local activity coherence were noted in the right nucleus accumbens, left intermediate reticular nucleus, and right hippocampal CA3 region. These results suggest that NaB alleviates the degree of inflammation in IBD mice and induces alterations in brain structure and function via the GBA.

## 1. Introduction

Inflammatory bowel disease (IBD) is a group of recurrent systemic inflammatory conditions characterised by symptoms including abdominal pain, diarrhoea, haematochezia and weight loss [1]. Traditionally, the prevalence of IBD was thought to correlate with regional levels of industrialisation; however, with the advancement of global industrialisation, IBD has progressively become a worldwide health concern [2]. IBD patients endure not only intestinal symptoms such as abdominal pain, diarrhoea and haematochezia, but also frequently experience extraintestinal manifestations, including anxiety and depression, significantly impairing quality of life. The gut–brain axis (GBA) represents a complex bidirectional communication network between the gastrointestinal system and the central nervous system, attracting considerable attention for its role in regulating neurological and metabolic health. The communication network between the gut and the central nervous system is intricate, encompassing the enteric nervous system, branches of the sympathetic and parasympathetic autonomic nervous system, and neuroimmune and neuroendocrine signalling pathways [3–5]. Visceral feedback from the gut to the spinal cord (specifically the thoracic and upper lumbar regions) and the solitary tract nucleus occurs via afferent spinal and vagal sensory nerves. Through this pathway, they connect with polysynaptic inputs to higher brain regions such as the hypothalamus and limbic forebrain. The cingulate and insular cortices, amygdala, bed nucleus of the stria terminalis and hypothalamus constitute regions modifying vagal and spinal autonomic outflow to visceral targets. The preganglionic autonomic projections from all these structures provide bidirectional control of the GBA [5, 6]. Beyond these neural pathways, hormones and humoral signalling molecules also participate in this communication [3–5].

Evidence suggests that psychological factors play a role in both the pathophysiology and disease course of IBD [7]. Alterations in the structure or function of distinct networks or circuits within the central nervous system, such as the limbic system and reward circuits, profoundly influence the emotional state and mental health of IBD patients [8, 9]. Consequently, investigating changes in brain structure and function among IBD patients holds significant value for assessing disease progression and mental wellbeing [10, 11]. MRI, with its advantages of high resolution, radiation‐free operation and multi‐parameter imaging capabilities, has become a vital tool for studying the brain structure and function. Structural MRI techniques, including voxel‐based morphological (VBM) analysis, diffusion tensor imaging (DTI) and diffusion kurtosis imaging (DKI), can assess changes in brain volume and the integrity of white matter tracts with high precision and reliability. Functional MRI (fMRI) enables non‐invasive, real‐time monitoring of neural activity changes in the brain during rest or specific tasks. It can reveal alterations in neural activity patterns within specific brain regions and functional connectivity abnormalities in IBD patients [12]. These changes may correlate with the patients’ psychological symptoms and the severity of intestinal inflammation. Consequently, fMRI provides a powerful tool for investigating alterations in brain function among IBD patients. Population‐based studies have identified structural or functional alterations in various brain regions among IBD patients, though results often exhibit considerable variability [9, 13, 14]. This discrepancy likely stems from differences in study samples, living conditions, and treatment regimens. Consequently, establishing animal models of IBD to evaluate brain structural and functional changes under diverse intervention conditions may yield more precise findings than those in human‐based research.

Among the microbial metabolites involved in GBA, short‐chain fatty acids (SCFAs) serve as key bioactive compounds, exerting a significant influence on both intestinal and cerebral functions. SCFAs, fatty acids with fewer than six carbon atoms, constitute a crucial metabolic product generated by the gut microbiota. Utilised by colonic cells as an energy source, they exert pronounced effects on intestinal homeostasis, energy metabolism and immune response regulation [15]. Previous studies have confirmed that SCFAs regulate the adaptive immune system by influencing macrophages and dendritic cells, as well as exerting direct effects on the adaptive immune system [16]. Butyrate, one of the most important SCFAs, serves as the primary energy source for colonic epithelial cells. Moreover, butyrate exhibits anti‐inflammatory properties, playing a crucial role in the treatment of IBD [13, 15]. Research indicates that butyrate can cross the blood–brain barrier, influencing neuroinflammatory responses and exhibiting potential neuroprotective effects on neural and brain cells, thereby alleviating anxiety and depression in IBD patients [17, 18]. Although butyrate can directly enter the brain, the quantity reaching it via the bloodstream is extremely limited. The majority of orally administered butyrate is consumed by intestinal cells as a primary energy source, while another portion is metabolised by the liver. Consequently, the dose ultimately entering systemic circulation and reaching the brain is minimal [15]. It indicates that butyrate, as a key SCFA, primarily influences the central nervous system of IBD patients via GBA when administered as a supplement or medication. However, the precise effects of butyrate supplementation on the brain function in IBD patients remain inconclusive. Investigating butyrate’s effects on the brain structure and function in IBD patients could elucidate its potential mechanisms in regulating GBA function, thereby providing novel theoretical foundations and intervention strategies for comprehensive IBD management. Currently, however, studies examining brain functional and structural alterations following butyrate supplementation in IBD populations remain scarce. Moreover, such studies often involve subjects who have already undergone multiple therapeutic interventions, introducing significant confounding factors that complicate the direct assessment of butyrate’s primary impact on brain function and structure. Correspondingly, animal studies in this area remain scarce.

This study used animal models and fMRI techniques to observe alterations in brain function and structure following sodium butyrate (NaB) supplementation in IBD mice. It analysed correlations with intestinal inflammatory markers to elucidate the effects of butyrate via GBA on brain structure and function in IBD mice.

## 2. Materials and Methods

Fifteen healthy 7‐week‐old BALB/c female mice were provided by the Tianjin Laboratory Animal Centre. All animals were randomly assigned to a healthy control group, an IBD model group, and an IBD + NaB group (NaB group). Five mice per group were housed in SPF‐grade animal facilities maintained at 25 ± 1°C and 50% humidity. They were fed standard dry rodent chow with free access to drinking water under a 12 h light/12 h dark cycle. Animal husbandry and experimentation adhered to Nankai University’s Animal Welfare Regulations (Ethics Approval Number: 2021‐SYDWLL‐000358). All animals underwent a 1‐week acclimatisation period before the experiment.

### 2.1. Establishment of the Murine IBD Model

Experiments commenced after a 1‐week acclimatisation period. Mice in the IBD and NaB groups were provided with a 3% dextran sulphate sodium (DSS) solution in place of drinking water for a 10‐day modelling period. Following the start of the experiment, mice were weighed daily, stool was collected for morphological examination and haematochezia was detected. From the experimental commencement date, the healthy control group and the IBD group received daily oral administration of autoclaved water. The NaB group received daily oral administration of NaB solution at a dose of 500 mg/kg according to body weight. All drinking water was replaced every other day.

### 2.2. Disease Activity Index (DAI) Scoring

To evaluate the severity of colitis, a DAI score was determined daily. The DAI is the combined score of weight loss, stool consistency and haematochezia. Scores were defined as follows [19]: weight loss: 0 (no loss), 1 (5%–10%), 2 (11%–15%), 3 (16%–20%) and 4 (>20%); stool consistency: 0 (normal), 2 (loose stool) and 4 (diarrhoea); and haematochezia: 0 (absence) and 4 (presence).

### 2.3. Observation of Intestinal Damage

At the end of the experiment, mice were anaesthetised via intraperitoneal injection of 1.25% Avodine at a dosage of 0.2 mL per 10 g of body weight. Following loss of consciousness, eyeballs were enucleated to collect peripheral blood, which was centrifuged at 2000 *r*/min for 10 min after standing at 4°C for 2 h. Serum was collected and stored at −80°C for subsequent use. Mice were then euthanised by cervical dislocation, and the large intestine was removed and its length measured. The intestinal contents were then flushed clean with pre‐chilled PBS. A segment of the large intestine was excised and immersed overnight in 4% paraformaldehyde solution, followed by paraffin block embedding and section preparation. Tissue sections were stained with haematoxylin and eosin (H&E) solutions, after which the structural morphology of the intestinal tissue was observed under an optical microscope. The scoring criteria are detailed in Supporting Information 1: Table S1.

### 2.4. Analysis of Serum Inflammatory Cytokine Levels

Using the mouse ELISA detection kit (MEIMIAN, Jiangsu, China), serum levels of IL‐6, IL‐1β and IL‐10 were measured according to the method described in the instruction manual.

### 2.5. MRI Acquisition

Following moulding completion, mouse functional magnetic resonance imaging data were acquired at the Multimodal Preclinical Molecular Imaging Centre of Tianjin Medical University General Hospital. A Bruker 9.4 T BioSpec 94/30 MRI & PET Insert in vivo small animal imaging system was employed, equipped with a 20 mm head surface coil. Anaesthesia comprised induction and maintenance phases. Mice were placed in an induction chamber containing a mixture of 3% isoflurane and 1% oxygen for induction anaesthesia. Once light reflexes were blunted and toe‐grip reflexes were absent, mice were transferred to the scanning bed. Following tracheal intubation, continuous anaesthesia was maintained using a mixture of 2.5% isoflurane and 0.5% oxygen delivered via a ventilator. The animal is secured in a prone position on a plastic board using a plastic cap and a jaw bar. Pressure sensors monitor the mouse’s respiratory rate and body temperature. Scanning commences once the respiratory rate is maintained at 80 breaths per minute and the body temperature stabilises. Throughout the scan, the animal’s body temperature, respiratory rate and heart rate amplitude are continuously monitored.

VBM analysis was used on three‐dimensional T2‐weighted imaging (3D T2WI). The 3D T2WI sequence utilising a rapid acquisition of relaxation enhanced (RARE) sequence. Parameters were as follows: repetition time (TR) = 1500 ms, repetition time (TE) = 38 ms, flip angle = 180°, sample matrix = 120 × 120, voxel size = 0.15 mm × 0.15 mm × 0.15 mm, 53 slices, slice thickness/gap = 0.15/0 mm. White matter fibre assessment was used DTI with diffusion weighting encoded along 30 independent directions, *b*‐value = 1000 s/mm^2^, sampling matrix = 80 × 80, field of view (FOV) = 100 mm × 100 mm and voxel size = 0.14 mm × 0.14 mm × 0.7 mm. fMRI functional imaging blood oxygen level dependent (BOLD) signal acquisition employed a T2star free‐echo‐planar echo sequence (T2star‐FID‐EPI), collecting 600 time points with parameters: TR = 1000 ms, TE = 14 ms, flip angle = 70°, sampling matrix = 53 × 53, voxel size = 0.3 mm × 0.3 mm × 0.6 mm, 25 slices and slice thickness/gap = 0.15/0 mm.

### 2.6. MRI Analysis

Preprocessing of the mouse 3D T2WI structural images commenced with manual brain segmentation using ITKSNAP software, followed by affine linear registration onto the Turone Mouse Brain Template and Atlas (TMBTA) anatomical template. Subsequently, based on the tissue probability map provided by the TMBTA template, the structural images were segmented into grey matter, white matter, and cerebrospinal fluid (CSF) images. Following segmentation, the structural images were spatially normalised to the template and subjected to Jacobian modulation to correct for volume changes. Finally, spatial smoothing was performed using a three‐dimensional Gaussian smoothing kernel with a full width at half maximum (FWHM) of 4 mm. The DTI image processing steps were as follows: first, images underwent eddy current correction and gradient direction rotation; the b0 image was extracted and a mask was created; registration was performed using T2‐weighted images (T2WI) and matched with the TMBTA template; and fractional anisotropy (FA) and mean diffusivity (MD) values were extracted for each white matter tract and brain region. The preprocessing steps for functional images are as follows: first, discard the initial 10 time points of resting‐state images; then, perform time‐layer correction and head motion correction; and subsequently, normalise functional images to the template using spatial transformation parameters derived from structural image normalisation. Subsequently, smoothing was performed using a three‐dimensional Gaussian kernel with a full width at FWHM of 4 mm. To mitigate interference from non‐neural signals, further detrending and noise covariate removal operations were conducted, followed by filtering.

Following preprocessing, VBM and fMRI images were further processed using SPM12 software (http://www.fil.ion.ucl.ac.uk). fMRI images were processed to derive the amplitude of low‐frequency fluctuations (ALFF) and regional homogeneity (ReHo) data. All computational and statistical analyses were performed using the SPM12 software. For VBM analysis, grey matter masks were generated from images of normal control mice. A one‐way ANOVA was conducted on the three mouse groups, using total intracranial volume (TIV) as a covariate, with a threshold set at *p*  < 0.001 and FDR correction applied. Following the acquisition of FA and MD values for each region of interest (ROI) from DTI data, statistical analysis and plotting were performed using RStudio. Statistical results were set at *p*  < 0.05 to indicate statistically significant differences, with data undergoing FDR correction. For ALFF analysis, whole‐brain signals underwent a fast Fourier transform to obtain power spectra, which were then square‐root extracted to isolate the low‐frequency amplitude (0.01–0.08 Hz). To eliminate inter‐individual variability, voxel‐specific ALFF values were divided by whole‐brain mean ALFF (mALFF) and standardised to yield the mALFF. Further z‐transformation produced zALFF values. Before spatial smoothing, Kendall’s coefficient of concordance (KCC) was calculated between each voxel’s time series and those of its 26 neighbouring voxels, assessing functional activity synchrony within the local region. To mitigate the influence of inter‐individual ReHo level variations, each voxel’s ReHo value was divided by the whole‐brain average ReHo value and standardised to yield the mean ReHo (mReHo). This was subsequently *z*‐transformed to produce zReHo values. Finally, the generated ReHo maps underwent spatial smoothing to enhance the signal‐to‐noise ratio (using a 4 mm smoothing kernel). Voxel‐based univariate analysis of variance was performed on zALFF and zReHo maps, with a threshold set at *p*  < 0.005 and FDR correction applied. Post‐hoc analysis of significantly differing brain regions was completed using RESTplus software (http://www.restfmri.net/forum/restplus).

### 2.7. Statistical Analysis

The statistical analysis of imaging results has been addressed in MRI analysis. All values are reported as the mean ± standard error of the mean (SEM). Full statistical details are listed in the figure legends. We used GraphPad Prism 10 software for all statistical analyses. Comparisons between the three groups were performed using one‐way ANOVA. Differences were considered statistically significant at *p*  < 0.05.

## 3. Results

### 3.1. NaB Ameliorates Intestinal Injury and Systemic Inflammation in IBD Mice

**Figure 1:**
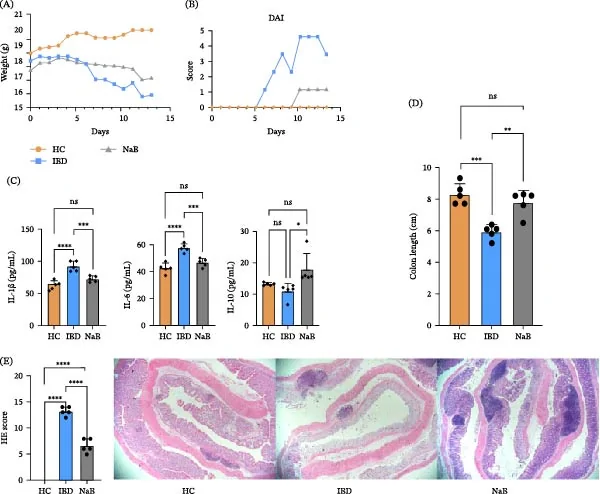
NaB influenced intestinal inflammation in IBD mice. IBD mice exhibited progressive weight loss over time following modelling, accompanied by increasing DAI scores, a trend mitigated by NaB administration (A, B). Serum inflammatory cytokine analysis revealed elevated pro‐inflammatory factors IL‐6 and IL‐1β alongside reduced anti‐inflammatory factor IL‐10 in the IBD group, with NaB reversing these alterations (C). Colon length in IBD mice was significantly shorter than in HC and NaB groups (D). H&E‐stained sections (4×) revealed markedly milder colonic damage in the NaB group compared to the IBD group (E). Data are presented as mean ± standard deviation (*n* = 5 per group). ns indicates no statistically significant difference;  ^∗^ *p*  < 0.05,  ^∗∗^ *p*  < 0.01,  ^∗∗∗^ *p*  < 0.001,  ^∗∗∗∗^ *p*  < 0.0001.

As the duration of IBD modelling increased, the body weight of the mice gradually decreased while the DAI score progressively rose; NaB mitigated this trend (all *p* < 0.05). Serum ELISA revealed elevated levels of pro‐inflammatory cytokines IL‐6 and IL‐1β in IBD mice compared to the HC group. NaB reduced pro‐inflammatory cytokine levels in IBD mouse serum while increasing anti‐inflammatory cytokine IL‐10 levels. Measurements of colon length revealed significant intergroup differences: 8.30 ± 0.67 cm in the HC group, 5.92 ± 0.46 cm in the IBD group, and 7.76 ± 0.76 cm in the NaB group. H&E staining demonstrated marked pathological alterations in the intestinal tissue of IBD mice compared to those in the HC group. In the HC group, intestinal glands were clearly defined with a regular arrangement, showing no significant inflammatory cell infiltration and intact villi without discernible disruption. The IBD and NaB groups exhibited varying degrees of intestinal wall oedema, crypt disruption, inflammatory cell infiltration and disorganised villi arrangement. The extent of intestinal wall damage in the NaB group was markedly less severe than that in the IBD group (Figure 1).

### 3.2. NaB Mice Show Reduced VBM Compared to IBD in the Left Caudoputamen

**Figure 2:**
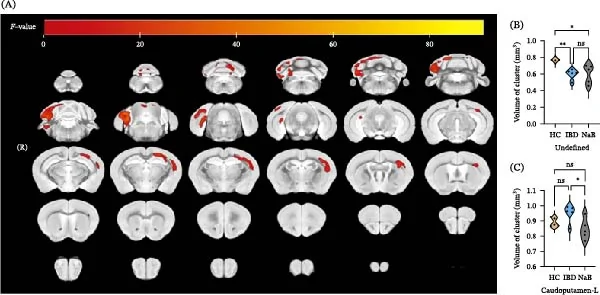
Differences in voxel‐level morphological features in specific brain regions across the three groups (A). VBM analysis revealed differences among the groups in two regions: an undefined region in the right cerebral hemisphere and the left caudoputamen. Within the undefined region, both the IBD and NaB groups exhibited reduced VBM compared to the HC group, whilst no statistically significant difference in brain volume was observed between the IBD and NaB groups. In the left caudoputamen, the NaB group demonstrated slightly smaller brain volume than the IBD group, though no significant statistical difference was noted relative to the HC group (B, C). ns indicates no statistically significant difference;  ^∗^ *p*  < 0.05,  ^∗∗^ *p*  < 0.01.

VBM analysis revealed differences among the HC group, IBD group, and NaB group in two regions: an undefined region in the right cerebral hemisphere and the left caudoputamen. Although the peak region within the undefined region lacks a specific statistical designation, this right‐hemispheric region encompasses multiple functional areas, including the dentate gyrus, hippocampal CA3 and parasubiculum. Within this region, both the IBD and NaB groups exhibited reduced VBM compared to the HC group, whilst no statistically significant difference in brain volume was observed between the IBD and NaB groups. In the left caudoputamen, the NaB group demonstrated a slightly smaller brain volume than the IBD group, though no significant statistical difference was noted relative to the HC group (Table 1 and Figure 2).

**Table 1:** VBM difference among the groups.

Peak region	Cluster size	Peak MNI coordinates			*F*‐value
		*x*	*y*	*z*	
Undefined	25,424	2.58	0.84	4.53	91.34
Caudoputamen‐L	10,908	−2.22	1.32	0.51	30.96

### 3.3. IBD and NaB Mice Showed Lower MD in the Left Inferior Cerebellar Peduncle When Compared to HC

All three groups measured the FA and mean diffusion (MD) values of 137 bilaterally symmetrical white matter tracts. Following FDR correction, the only significant MD difference between the three groups was observed in the left inferior cerebellar peduncle (restiform body) region. Specifically, the HC group exhibited higher MD values in this region compared to the other two groups, whilst no statistically significant difference was found between the latter two groups (Supporting Information 2: Figure S1).

### 3.4. NaB Modulates Abnormal Neural Activity in Specific Brain Regions of IBD Mice

**Figure 3:**
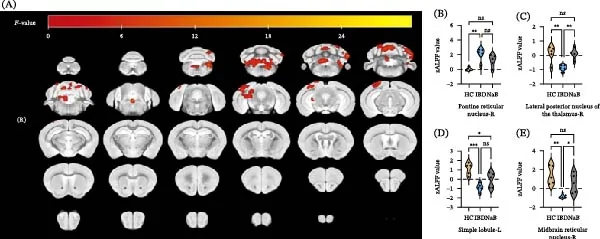
Differences in neural activity were observed across the three groups (A). zALFF comparisons among the groups revealed that, compared with the HC group, the IBD group exhibited significantly heightened neural activity in the right pontine reticular nucleus (B). Conversely, reduced neural activity was observed in the right lateral posterior nucleus of the thalamus, left simple lobule, and right midbrain reticular nucleus (C–E). In contrast to the IBD group, the NaB group showed heightened neural activity in the right lateral posterior nucleus of the thalamus and the right midbrain reticular nucleus, displaying no statistically significant differences relative to the HC group in these regions (C, E). However, no significant differences in neural activity alterations within the right pontine reticular nucleus and left cerebellar simple lobule were observed between the NaB and IBD groups (B, D). ns indicates no statistically significant difference;  ^∗^ *p*  < 0.05,  ^∗∗^ *p*  < 0.01,  ^∗∗∗^ *p*  < 0.001.

zALFF comparisons among the groups revealed that, compared with the HC group, the IBD group exhibited significantly heightened neural activity in the right pontine reticular nucleus. Conversely, reduced neural activity was observed in the right lateral posterior nucleus of the thalamus, left simple lobule and right midbrain reticular nucleus. In contrast to the IBD group, the NaB group showed heightened neural activity in the right lateral posterior nucleus of the thalamus and the right midbrain reticular nucleus, displaying no statistically significant differences relative to the HC group in these regions. However, no significant differences in neural activity alterations within the right pontine reticular nucleus and left cerebellar simple lobule were observed between the NaB and IBD groups (Table 2 and Figure 3).

**Table 2:** zALFF and zReHo difference nuclei among three groups.

Peak region	Cluster size	Peak MNI coordinates			*F*‐value
		*x*	*y*	*z*	
zALFF					
Pontine reticular nucleus‐R	360	0.12	0.12	−3.51	10.65
Lateral posterior nucleus of the thalamus‐R	205	1.92	2.32	−1.91	9.87
Simple lobule‐L	170	−3.08	1.32	−5.11	12.64
Midbrain reticular nucleus‐R	154	0.12	2.32	−4.51	9.763
zReHo					
Nucleus accumbens‐R	132	1.52	−1.28	1.09	11.74
Intermediate reticular nucleus‐L	265	−0.88	−0.68	−5.31	15.71
Field CA3‐R	238	3.32	1.12	−2.31	15.41

### 3.5. NaB Modulated Alterations in Local Activity Coherence Within Specific Brain Regions of IBD Mice

**Figure 4:**
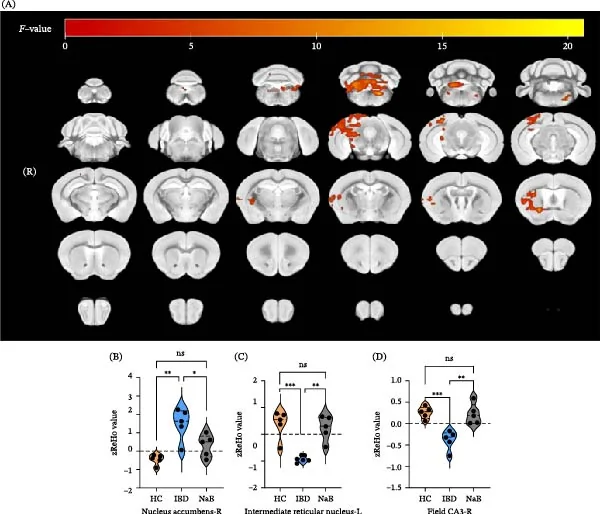
Differences in neural local activity coherence were observed across the three groups (A). zReHo comparisons among the groups revealed that, relative to the HC group, the IBD group exhibited increased local activity coherence in the right nucleus accumbens, while the NaB group showed a reversal of this effect, aligning more closely with the HC group (B). In contrast, the IBD group displayed decreased coherence in the left intermediate reticular nucleus and right hippocampal CA3 region relative to the HC group (C, D). This decreasing trend was absent in the NaB group, which showed no marked deviations from the HC group in these regions (C, D). ns indicates no statistically significant difference;  ^∗^ *p*  < 0.05,  ^∗∗^ *p*  < 0.01,  ^∗∗∗^ *p*  < 0.001.

zReHo comparisons among the groups revealed that, relative to the HC group, the IBD group exhibited increased local activity coherence in the right nucleus accumbens, while the NaB group showed a reversal of this effect, aligning more closely with the HC group. In contrast, the IBD group displayed decreased coherence in the left intermediate reticular nucleus and right hippocampal CA3 region relative to the HC group. This decreasing trend was absent in the NaB group, which showed no marked deviations from the HC group in these regions (Table 2 and Figure 4).

## 4. Discussion

SCFAs play a crucial role in maintaining intestinal epithelial barrier function and immune homeostasis. Butyrate, in particular, engages multiple signalling pathways within intestinal immune cells and epithelial cells to restore compromised colonic barrier function and gut homeostasis [20]. In IBD patients, secretion of inflammatory cytokines such as IL‐1, IL‐6, IL‐17 and TNF increases [21]. Previous studies have confirmed that NaB reduces the levels of pro‐inflammatory factors in serum from IBD mice while promoting the release of anti‐inflammatory factors, thereby alleviating intestinal inflammation [22–24]. These conclusions align with the findings of the present study. In this investigation, NaB supplementation mitigated DSS‐induced intestinal inflammation, consistent with prior research outcomes.

Previous studies on GBA in IBD mice have often relied on behavioural testing to assess alterations in the central nervous system, failing to provide direct insights into changes in brain structure and function. However, with advancements in imaging equipment and algorithmic updates, brain MRI has emerged as an effective tool for objectively evaluating structural and functional alterations in the brains of IBD mice. Numerous prior investigations have demonstrated that NaB influences the central nervous system via the GBA [25–28]. This study employed VBM and DTI to separately evaluate grey and white matter alterations in IBD mice, assessing NaB’s impact on their brain structure. VBM comparisons revealed differences in an undefined region in the right cerebral hemisphere and the left caudoputamen between the HC, IBD and NaB groups. Although the peak region within the undefined area lacks a specific statistical designation, this right‐hemispheric region encompasses multiple functional areas, including the dentate gyrus, hippocampal CA3 and parasubiculum. These regions participate in emotional regulation. Within these areas, both the IBD and NaB groups exhibited reduced VBM compared to the HC group. However, no statistically significant difference in brain volume was observed between the IBD and NaB groups in this region. This indicates that IBD induces structural abnormalities in emotion‐related brain areas, specifically manifested as reduced volume in the corresponding regions. This may be associated with neuronal degeneration leading to cellular atrophy, a change that NaB failed to ameliorate. Within the left caudoputamen, the NaB group exhibited a slightly reduced volume compared to the IBD group, though no significant difference was observed relative to the HC group. This finding may reflect localised brain tissue swelling due to cytotoxic oedema or represent a compensatory adaptation. Previous studies have identified enlarged volumes in the amygdala, hypothalamus, putamen and pallidus nuclei of IBD patients compared to healthy controls, which are also implicated in neurodegeneration [13]. Prior research indicates that NaB suppresses neuroinflammation by correcting microglial polarisation associated with the GPR109A/PPAR‐γ/TLR4‐NF‐κB pathway [25]. Neuroinflammation induces glial proliferation and activation, releasing inflammatory cytokines that precipitate cytotoxic oedema. As neuroinflammation intensifies, synaptic loss, mitochondrial dysfunction and abnormal protein aggregation ensue, triggering heightened inflammatory responses and neuronal death, ultimately resulting in nucleus atrophy. In this study, the divergent trends in nucleus volume across regions among the three groups may correlate with the severity of neuroinflammation. Specifically, MD values in the left inferior cerebellar peduncle (restiform body) differed significantly between groups, with reduced MD values observed in both the IBD and NaB groups. MD values reflect the degree of freedom in water molecule diffusion within the ROI; decreased MD indicates restricted water diffusion within the corresponding area. This discrepancy may also stem from localised cytotoxic oedema, altering water diffusion within fibre tracts. Within white matter tracts, MD changes often correlate with FA alterations; however, no significant statistical intergroup differences in FA values were observed across the studied brain regions. This inconsistency undermines the reliability of the MD differences between the three groups. It is also possible that NaB did not significantly affect the DTI metrics in this experiment. Consequently, further research with larger sample sizes is warranted to validate these findings.

This study also employed fMRI to investigate the effects of NaB on the GBA in IBD mice. Findings revealed heightened neural activity in the right thalamic reticular nucleus region of IBD mice compared to that of HC mice. Activation in this area may correlate with heightened vigilance, a feature observed in anxiety disorders. Previous research has implicated abnormal activity in the reticular nucleus region with autistic symptoms [29], suggesting that increased neural activity in this region of IBD mice may contribute to their emotional abnormalities. Furthermore, compared to HC mice, IBD mice exhibited reduced neural activity in the right lateral posterior nucleus of the thalamus, left simple lobule and right midbrain reticular nucleus. Abnormalities in these thalamic and cerebellar regions may contribute to motor impairments observed in mice. Previous studies on the mouse GBA predominantly employed behavioural tests to assess emotional state alterations. Results from the water maze tests and open field tests may reflect not only cognitive function but also motor capabilities. Whether the reduced activity observed in the lateral posterior nucleus of the thalamus and cerebellar simple lobule in this study actually impacts motor function requires validation through larger‐scale animal experiments. However, the possibility that damage to these nuclei could exert an effect cannot be ruled out. In this study, altered neural activity in the midbrain reticular nucleus and the pontine reticular nucleus exhibited opposing trends. This discrepancy may arise because the anatomically centred regions calculated for these nuclei do not encompass their entirety; these areas may overlay other functionally distinct nuclei that also contribute to neural alterations. In this study, NaB elevated neural activity in the right lateral posterior nucleus and midbrain reticular nucleus of IBD mice, suggesting that NaB may alter mood and motor function in IBD mice via the GBA. Analysis of ReHo values across three mouse groups revealed elevated zReHo values in the right ventral tegmental area of IBD mice, alongside reduced zReHo values in the hippocampal CA3 region. This reflects abnormalities in the reward circuitry of IBD mice, a trend reversed by NaB. The opposing trends in zReHo values between the nucleus accumbens and hippocampus in IBD mice may relate to neuroinflammation. Inflammation within the nucleus accumbens activates microglia, releasing pro‐inflammatory factors. These factors enhance functional connectivity between neurones by activating TLR4 receptors on accumbens neurones. Conversely, neuroinflammation suppresses hippocampal neurogenesis, reducing neuronal synchrony [30, 31]. The diminished neuronal synchrony in the reticular nucleus of the midbrain in IBD mice suggests potential motor dysfunction. The effects of NaB on the central nervous system of IBD mice primarily manifest through alterations in the neuronal function. It is noteworthy that in this study, many of the differentially affected nuclei encompassed regions partially overlapping with the cerebellum. Given the limited prior research on cerebellar function, the structural and functional alterations observed in these areas warrant further validation and analysis. However, recent years have seen increasing attention paid to the role of cerebellar function in neurological disorders. Our findings may prompt other researchers to focus on cerebellar functional alterations in IBD patients, with future studies likely to yield deeper insights into these mechanisms [32].

This study employed MRI technology to objectively assess the impact of NaB on the brain structure and function in IBD mice. It enabled the effects of NaB to be refined to the level of functional brain regions, thereby aiding our deeper understanding of NaB’s influence on GBA. Furthermore, the observation that different brain regions exhibited distinct patterns of structural and functional changes may stem from varying stages of neuronal injury. This finding also contributes to our comprehension of the phased alterations in brain damage induced by IBD. This research may be extended to humans to investigate the effects of NaB on the brain structure and function in IBD patients. Future applications could provide therapeutic insights for individuals with diverse emotional disorders.

This study has several limitations. The sample size was relatively small, and all experimental mice were female. Changes in oestrogen levels during the experiment may have influenced the findings regarding brain function. This study did not include groups receiving different doses or administered at different timescales; therefore, it was not possible to assess the effects of NaB at different concentrations and with different reaction times on GBA in IBD mice. However, as this study is a preliminary investigation, we designed the experiment such that the NaB administration cycle coincided with the IBD modelling cycle; we believe this design reflects the effects of NaB on intestinal and brain function in IBD mice. Of course, model studies involving different doses and different administration timescales would be highly valuable, and this is an area we intend to refine in future work. Furthermore, this study did not include additional basic experimental analyses to elucidate the specific immune mechanisms underlying differences across distinct brain regions, preventing a clear understanding of how NaB induces these nuclear alterations. Our team will subsequently conduct larger‐scale animal studies, comparing the effects of IBD on brain structure and function in female versus male mice, alongside experiments investigating immune pathways. This will aim to elucidate further the impact of NaB on GBA in IBD mice.

## 5. Conclusion

This study innovatively employed fMRI to evaluate the effects of NaB on GBA in IBD mice, revealing that NaB possesses anti‐inflammatory properties capable of alleviating inflammation in IBD mice. Furthermore, NaB influences alterations in the brain structure and function in IBD mice, a phenomenon potentially linked to its capacity to modulate the degree of neuroinflammation. The effects of sodium butyrate on the gut and brain of IBD mice may provide a basis for future drug development aimed at delaying or preventing long‐term neurological complications associated with IBD.

## Funding

This work was partially sponsored by the National Natural Science Foundation of China (Grant 12174203), the Tianjin Union Medical Center Project (Grant 2020YJ023), the China Postdoctoral Science Foundation (Grant 2021M702461) and the Tianjin Medical University Integrated Traditional Chinese and Western Medicine Research Fund (Grant 2024XKZXY08).

## Conflicts of Interest

The authors declare no conflicts of interest.

## Supporting Information

Additional supporting information can be found online in the Supporting Information section.

## Supporting information


**Supporting Information 1** Table S1. Histopathological scoring critical.

**Supporting Information 2** Figure S1. Heatmaps of FA values and MD values across three groups, with intergroup differences observed solely in the left inferior cerebellar peduncle (restiform body) region.

## Data Availability

The experiment data are available from the corresponding author upon reasonable request.
