# Supplementary material for: Investigating the Role of Sodium Butyrate in Mouse Models of IBD: A Multimodal Structural and Functional MRI Study
Source: Neural Plast. 2026 Sep 9;2026:9003986. doi: 10.1155/np/9003986 (PMC13555390; doi:10.1155/np/9003986)
Supplement: Supplementary file 1 [file NP-2026-9003986-s002.docx]

**Supplementary Table 1.** Histopathological scoring critical

| Score | Severity of inflammation | Crypt damage | Ulceration |
| --- | --- | --- | --- |
| 0 | Rare inflammatory cells in the lamina propria | Intact crypt | 0 foci of ulceration |
| 1 | Increased numbers of granulocytes in the lamina propria | Loss of basal 1/3 of crypt | 1–2 foci of ulceration |
| 2 | Confluent inflammatory cells extended to submucosa | Loss of basal 2/3 of crypt | 3–4 foci of ulceration |
| 3 | Transmural extension of the inflammatory infilitration | Loss of entire crypt | Confluent or extensive ulceration |
| 4 | (-) | Change in epithelial surface caused by erosion | (-) |
| 5 | (-) | Confluent erosion | (-) |

**Supplementary Figure 1.** Heatmaps of FA values and MD values across three groups, with intergroup differences observed solely in the left inferior cerebellar peduncle (restiform body) region.
